# Two new taxa of Gesneriaceae in the karst regions in North Vietnam

**DOI:** 10.3897/phytokeys.157.54697

**Published:** 2020-08-26

**Authors:** Wen-Hong Chen, Shi-Wei Guo, Hieu Quang Nguyen, Li Chen, Yu-Min Shui

**Affiliations:** 1 CAS Key Laboratory for Plant Diversity and Biogeography of East Asia, Kunming Institute of Botany, Chinese Academy of Sciences, 132 Lanhei Road, Kunming 650201, Yunnan, China Chinese Academy of Sciences Kunming China; 2 Center for Plant Conservation of Vietnam (CPC), Vietnam Union of Science and Technology Associations, 25/32 Lane 191, Lac Long Quan Rd., Hanoi, Vietnam Karst Conservation Initiative of Yunnan Kunming China; 3 School of Life Sciences, Yunnan University, Kunming 650091, Yunnan, China University of the Chinese Academy of Sciences Beijing China; 4 University of the Chinese Academy of Sciences, Beijing 100049, China Vietnam Union of Science and Technology Associations Hanoi Vietnam; 5 Karst Conservation Initiative of Yunnan, Kunming 650201, Yunnan, China Yunnan University Kunming China

**Keywords:** Didymocarpoideae, Flora of Vietnam, limestone, natural park, new taxa, protection

## Abstract

One new species and one new variety of Gesneriaceae in Vietnam are described, viz. Paraboea sinensis var. glabrissima**var. nov.** and *Primulina xuansonensis***sp. nov.** These two new taxa grow in limestone regions in North Vietnam. The former new variety differs from Paraboea sinensis (Oliv.) Burtt var. sinensis in its young leaf abaxially, stem and peduncle sparsely and thin pannose, acute top of leaves, pistil glandular-pubescent or pubescent. The latter new species differs in its bracts 1.6–2.5 × 1.3–1.5 cm, corolla 3–3.5 cm long, corolla tube slightly curved near the base and inflated on the adaxial surface near the corolla lobes, and corolla abaxial lip lobes rounded. The two new taxa grow at Xuan Son National Natural Reserve, North Vietnam and remain undisturbed with low risk of extinction.

## Introduction

Vietnam is one of the regions with the highest species biodiversity in Asia (Myers et al. 2000). It is estimated that there are about 70 species of Gesneriaceae in Vietnam (Ho 2000). However, biodiversity studies are scarce in Vietnam. For example, the discoveries in Gesneriaceae only focused on certain genera: *Oreocharis* in North Vietnam (Do et al. 2016, 2017; Chen et al. 2017, 2018a; Möller et al. 2018), *Billolivia* in Central Vietnam (Middleton et al. 2014a, 2014b; Luu et al. 2015, 2018; Vu et al. 2015; Nguyen et al. 2016; Lý 2017) and other genera (*Deinostigma*: Möller et al. 2016; *Didymocarpus*: Hong et al. 2018; *Raphiocarpus*: Vu et al. 2012; *Hemiboea*: Chen et al. 2018b; *Paraboea*: Middleton 2018; *Aeschynanthus*: Middleton and Atkins 2018; and so on).

**Figure 1.**
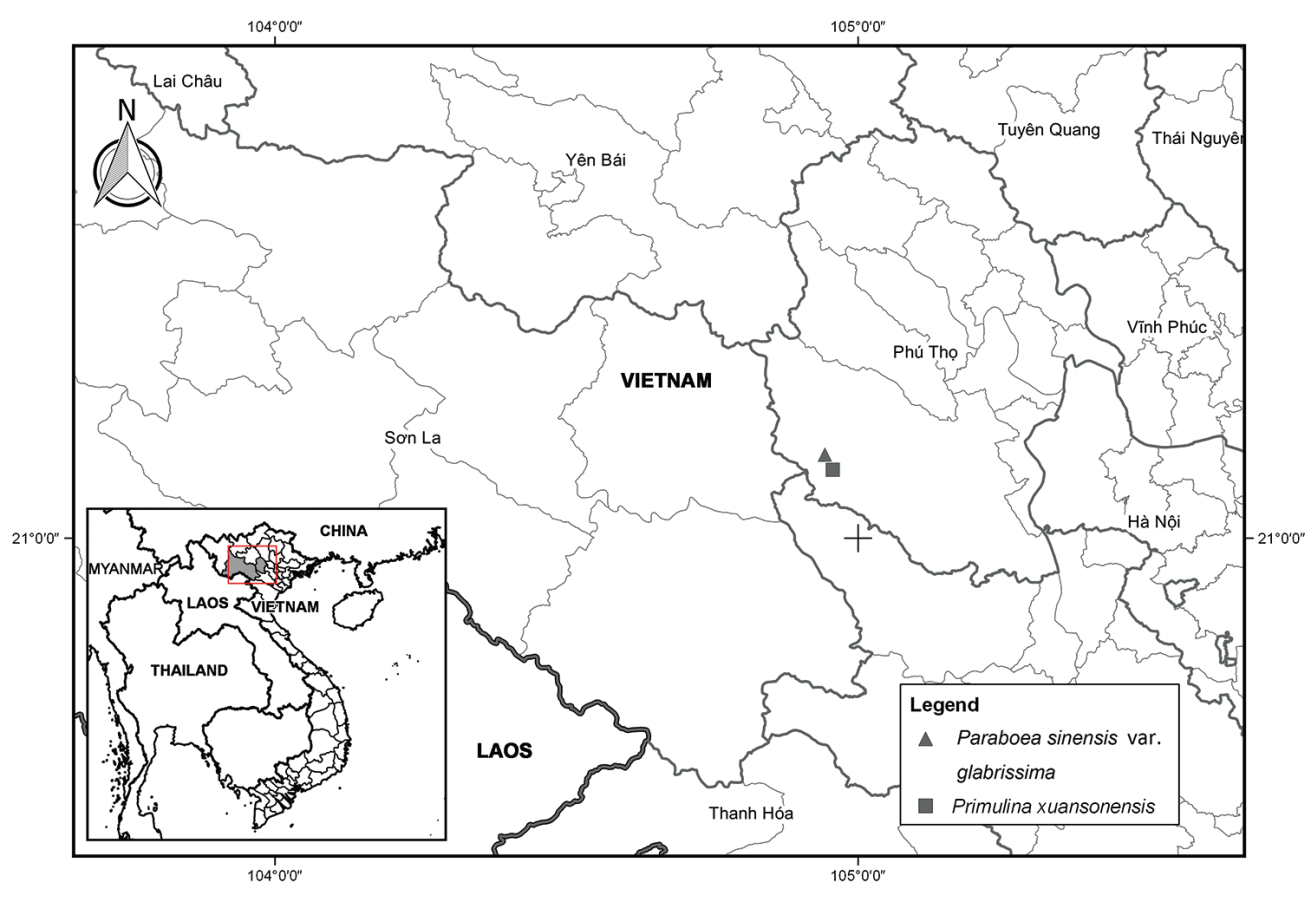
The distribution of Paraboea sinensis var. glabrissima W.H.Chen & Y.M.Shui var. nov. (triangle) and *Primulina xuansonensis* W.H. Chen & Y.M. Shui sp. nov. (square).

Many species of Gesneriaceae are distributed both in South China and in North Vietnam. China is a significant centre of diversity of the family Gesneriaceae, with the majority of taxa found in the South and Southwest China (Wang et al. 1990, 1998; Li 1991, 2005; Wei et al. 2010; Möller et al. 2016). After long-term collaborative surveys between China and Vietnam, we not only confirmed the common species and records from two countries, but also published some new species endemic to Vietnam, especially in the adjacent region to South China (Chen et al. 2017, 2018a, 2018b). Here, we describe two new taxa of Gesneriaceae in the karst regions at Xuan Son National Park in North Vietnam (Fig. 1), viz. Paraboea sinensis var. glabrissima W.H.Chen & Y.M.Shui, var. nov. and *Primulina xuansonensis* W.H.Chen & Y.M.Shui, sp. nov.

## Materials and method

After reviewing preserved herbarium specimens worldwide (BM, E, HNU, K, KUN, P, PE), two new taxa were confirmed. Habitat photographs and images of macro-morphological characters were taken in the field, in greenhouses and from the fixed (FAA) material. Morphological observations and measurements of the new taxa, based on living and dry plant specimens (from KUN) and preserved materials, were carried out. All morphological characters were observed and photographed with a Leica S8 APO stereomicroscope and a Nikon D700 microscope camera.

## Taxonomy

### Paraboea sinensis var. glabrissima

Taxon classificationPlantaeLamialesGesneriaceae

W.H.Chen & Y.M.Shui var. nov.

4B779998-25FC-505B-9728-8CF823CA656D

urn:lsid:ipni.org:names:77211199-1

Figure 2

#### Type.

Vientam, Pho Tho Province: Xuan Son County, Xuan Son National Park, 21°07'49.3"N, 104°57'09"E, 463 m a.s.l., 8 April 2016, *Y. M. Shui*, *W.H. Chen*, *C. Liu*, *H.Q. Nguyen*, *H.T. Nguyen*, *N.Q. Chuong CK909* (holotype, KUN!; isotype, CPC!=Herbarium of Center for Plant Conservation of Vietnam).

#### Diagnosis.

The new variety is similar to Paraboea sinensis (Oliv.) Burtt var. sinensis in its morphology of habit, calyx, corolla and fruits, but differs in its young leaf abaxially, stem and peduncle sparsely and thin pannose (vs. dense and thick pannose), acute top of leaves (vs. acuminate), pistil glandular-pubescent or pubescent (vs. glabrous).

Subshrubs. Stem erect, 50–80 cm tall, ca. 0.3 cm in diam., with many branches, pannose when young. Leaves opposite, equal to subequal in pairs on the stem; petiole 1–10 cm long, pannose when young; blade herbaceous, slightly asymmetric, oblong to obovate, 9–19 × 3.5–8 cm, base cuneate, apex acute, margin denticulate from the base, adaxially glabrous, abaxially sparsely pannose when young; venation penninerved, lateral veins 7–12 on each side of the midrib. Cymes axillary near branch apices; peduncle 1.5–4.5 cm long, sparsely pannose; bracts caducous; pedicel 1.4–2 cm long, sparsely pannose; bracteoles caducous. Calyx zygomorphic, 2-lipped, adaxial calyx ca. 1 cm long, 3-lobed to the middle, lobes rounded ca. 0.5 × 0.5 cm, abaxial calyx 2-lobed to the base, lobes obovate, ca. 1.2 × 0.6 cm, apex round, margin entire, outside glabrous, inside glabrous. Corolla campanulate, zygomorphic, 2–2.3 cm long, ca. 1.3 cm wide at the throat, both sides glabrous, tube 1–1.4 cm long; limb 2-lipped; adaxial lip 2-lobed, lobes broadly ovate, ca. 0.9 × 1.5 cm; abaxial lip 3-lobed, lobes broadly ovate, middle lobe ca. 0.7 × 1.2 cm. Stamens 2, adnate to the corolla base; anthers glabrous; filaments ca. 1 cm long, pubescent, staminodes 3, glandular-pubescent, lateral ones 2.5–3.0 mm long, adnate to the corolla tube base, the middle one ca. 1 mm long, adnate to the corolla tube ca. 2 mm above the base. Disc absent. Pistil ca. 1.2 cm long; ovary linear, ca. 0.8 cm long, sparsely glandular-pubescent or pubescent; style linear, ca. 0.4 cm long, glandular-pubescent; stigma 1, capitate. Capsule linear, spirally twisted, 2.5–3.7 cm long, glabrous.

**Figure 2.**
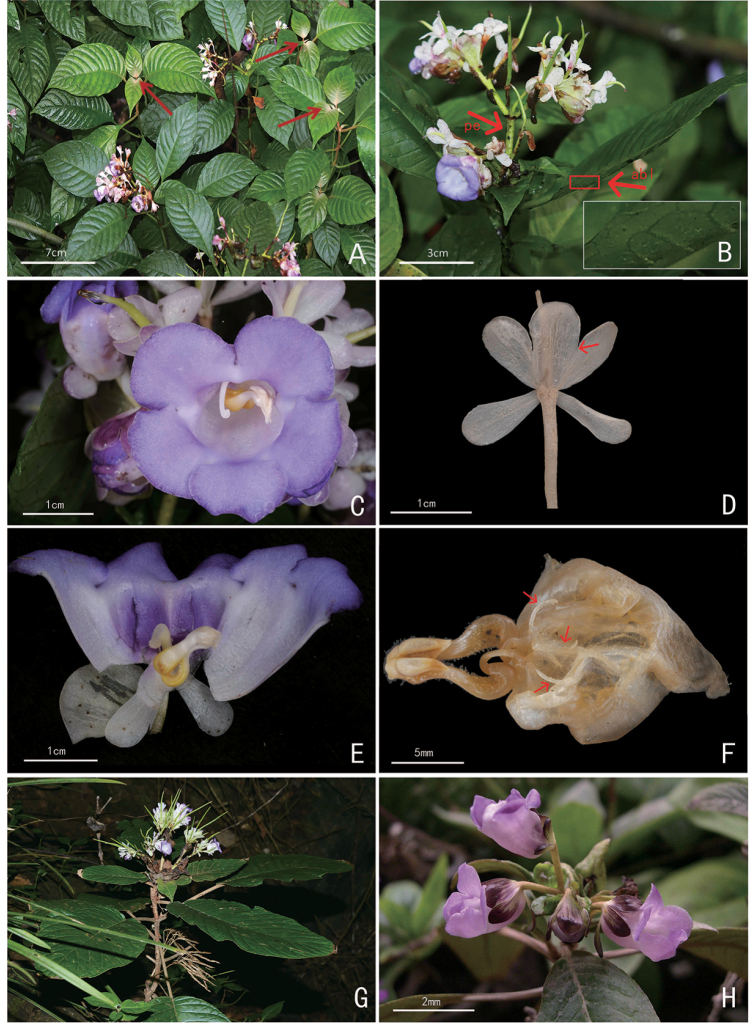
Paraboea sinensis var. glabrissima W.H.Chen & Y.M.Shui, var. nov. (**A–F**) and P. sinensis (Oliv.) Burtt var. sinensis (**G & H**) **A** habit, arrows indicate the young leaves with thin brown pannose **B** inflorescence with twisted fruits, arrows showing the indumentum of abaxial surface of leaf (pe = peduncle, abl = abaxial leaf) **C** face view of flower **D** dorsal view of the calyx, the arrow showing the coherent position **E** birds-eye view of opened corolla **F** stamens and staminodes, arrows showing the staminodes **G** habitat **H** inflorescence.

#### Phenology.

Flowering from March to April, fruiting from April to May.

#### Etymology.

The word “*glabrissima*” indicates the glabrous surface across the whole plant except the young leaves (Fig. 2A), which become glabrous as soon as the leaves become mature. In this manner, it is different from the original variety of Paraboea sinensis var. sinensis.

#### Distribution and habitat.

The new variety only occurs in Xuan Son County, Pho Tho Province and grows in soil, on rocks in limestone forests.

#### Provisional conservation status.

Based on field surveys and detailed discussions with Vietnamese colleagues, including Hieu Quang Nguyen, this new variety has only been observed in the Xuan Son National Park. There were only a total of 30 mature individuals, so we provisionally considered it as Critically Endangered (CR): B1b (v) + 2b (v). (IUCN 2012; IUCN Standards and Petitions Subcommittee 2017).

#### Note.

This new variety is very similar to the original variety of *Paraboea sinensis* in subshrub habit, obovate calyces and twisted fruits, but different in the almost glabrous habit (Table 1; Wang et al. 1998; Xu et al. 2008). Furthermore, the new variety is covered by pannose indumenti on the young leaves (Fig. 1A), but quickly becomes glabrous on the mature ones, while pannose indumenti consistently covers the original variety on both young and mature leaves (Figs 1G, H). Additionally, the top of the leaf is acute rather than acuminate in the original variety. It is necessary to explore the morphology diversity and genetic differentiation of *Paraboea sinensis* in the future, a wide-distributed species in the genus.

**Table 1.** Morphological comparison of Paraboea sinensis var. glabrissima W.H. Chen & Y.M. Shui and P. sinensis var. sinensis.

Characters	P. sinensis var. glabrissima	P. sinensis var. sinensis
Indumenti	Young leaf abaxially, stem and peduncle sparsely and thin pannose, mature glabrous	Young and mature leaf abaxially, stem and peduncle dense and thick pannose
Base of leaves	Cuneate	Broadly cuneate to round
Top of leaves	Acute	Acuminate
Pistil	Glandular pubescent or pubescent	Glabrous

### Primulina xuansonensis

Taxon classificationPlantaeLamialesGesneriaceae

W.H.Chen & Y.M.Shui sp. nov.

E5FC5358-B6A1-5690-8C45-AB42F71D81A9

urn:lsid:ipni.org:names:77211200-1

Figure 3

#### Type.

Vientam. Phu Tho Province: Xuan Son County, Xuan Son National Park, 21°07'01"N, 104°57'29"E, 438 m a.s.l., without flowers and fruits, 8 April 2016, introduced at Kunming Botanical Garden, in flower from October to December 2017, *Y. M. Shui, W.H. Chen, C. Liu, H.Q. Nguyen, H.T. Nguyen, N.Q. Chuong B2017-1341* (holotype, KUN!; isotype, CPC!).

#### Diagnosis.

The new species is similar to *Primulina lungzhouensis* (W.T. Wang) Mich.Möller & A.Weber in having prominent bracts, with a corolla, the outside of which is white. However, it differs in its bracts 1.6–2.5 × 1.3–1.5 cm (vs. bracts 2.5–3.8 × 2.2–2.8), corolla 3–3.5 cm long (vs. corolla 4.5–5 cm long), corolla tube slightly curved near the base (vs. not curved), inflated on the adaxial surface near the corolla lobes (vs. not inflated) and corolla abaxial lip lobes rounded (vs. triangular).

Herbs perennial. Stem absent. Leaves basal; petiole 2–5 cm long, strigose; blade carnose and papyraceous when dry, ovate to rounded, 11–18 × 8.5–11 cm, base cuneate, apex round, margin crenate from the base, adaxially sparsely strigose, abaxially sparsely strigose; venation penninerved, lateral veins 5–7 on each side of the midrib. Cymes umbel-like, axillary, ca. 9-flowered; peduncle 6.5–8.5 cm long, densely strigose; bracts 2, triangular-ovate, 1.6–2.5 × 1.3–1.5 cm, both sides densely strigose, margin with ca. 2-minute dentes near apex; pedicel 1–1.2 cm long, glandular-pubescent; bracteoles 2, narrowly ovate, ca. 1.2 × 0.4 cm, both sides densely strigose. Calyx 5-parted to the base, segments lanceolate, 0.8–1 × ca. 0.3 cm; apex acute, margin entire, outside glandular-pubescent, inside strigose. Corolla funnelform, zygomorphic, 3–3.5 cm long, ca. 0.8 cm wide at the throat, outside white, densely glandular-pubescent, inside white with two yellow stripes along the abaxial lip, glabrous; tube 2–2.3 cm, slightly curved near the base; limb 2-lipped; adaxial lip 2-lobed, lobes broadly ovate, ca. 0.5 × 0.8 cm; abaxial lip ca. 1.2 × 1.7 cm, 3-lobed to the middle, lobes rounded, middle lobe ca. 0.8 × 0.7 cm, lateral lobes ca. 0.6 × 0.7 cm. Stamens 2, adnate to the abaxial side of corolla tube ca. 1.4 cm above base; anthers glabrous; filaments glabrous, ca. 0.5 cm long; staminodes 3, ca. 1 mm long, glabrous, lateral two adnate to ca. 1 cm above the corolla base, central staminode adnate to ca. 0.6 cm above the corolla base. Disc ring-like, 1–2 mm high. Pistil glandular-pubescent, ca. 1.6 cm long; ovary linear, ca. 1 cm long; style linear, ca. 0.6 cm long; stigma cuneate, 2-lobed. Capsule unknown.

**Figure 3.**
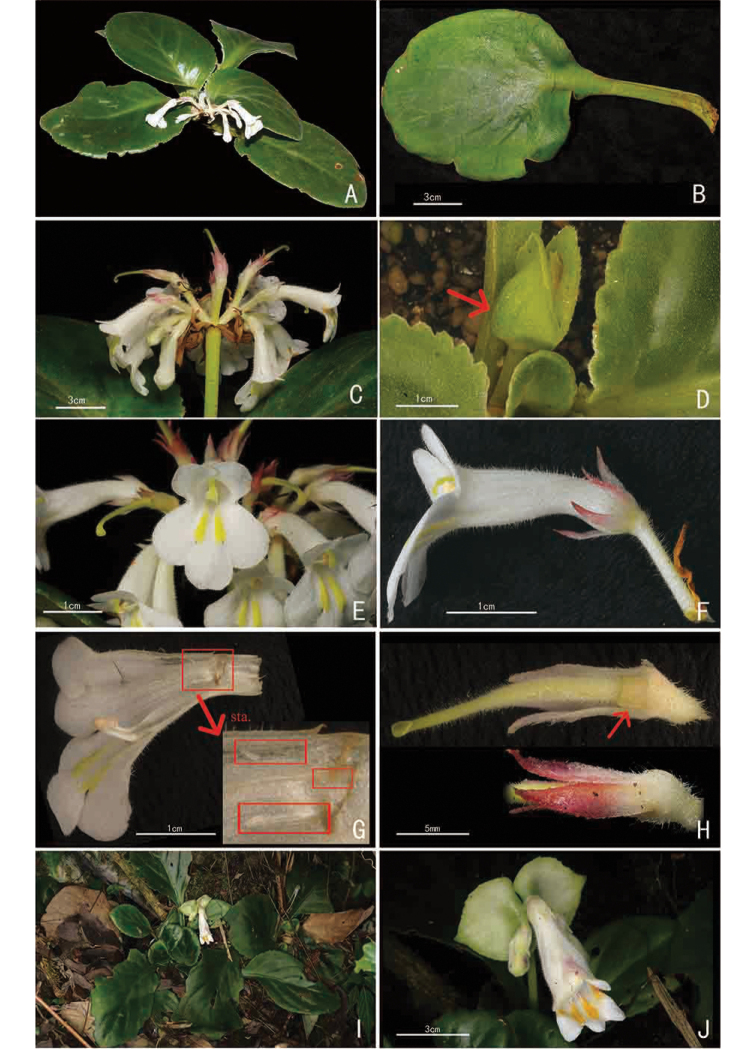
*Primulina xuansonensis* W.H.Chen & Y.M.Shui, sp. nov. (**A–H**) and its similar species *P. lungzhouensis* (W.T.Wang) Mich.Möller & A.Weber (**I–J**) **A** habit **B** view of adaxial leaf **C** inflorescence **D** bracts **E** face view of flower **F** lateral view of flower **G** birds-eye view of the opened corolla, arrow showing the staminodes (sta.) **H** pistil and calyx, arrow showing the disc ring **I** habitat of *P. lungzhouensis***J** face view of flower and bracts.

#### Phenology.

Flowering from October to December, fruiting from December to January from cultivated plants.

#### Etymology.

The name refers to the type locality.

#### Distribution and habitat.

The new species only grows in the limestone forests’ rock crevices and distributes in the type locality, Xuan Son National Park of Pho Tho Province, North Vietnam.

#### Provisional conservation status.

We observed it in the field with very few individuals (about ten individuals). Due to the similarity of plants without flowers in the genus *Primulina*, we did not pay attention to the species in the field, so the number of mature individuals remains unknown. Provisionally, it is best to consider it as Data Deficient (DD) (IUCN 2012; IUCN Standards and Petitions Subcommittee 2017).

#### Note.

Within the genus *Primulina*, there is a morphological species complex with prominent shell-like bracts, such as *P. beiliuensis* B. Pan & S. X.Huang, *P. eburnea* Hance, *P. lunglinensis* (W.T. Wang) Mich. Möller & A.Weber, *P. lungzhouensis*, *P. maguanensis* (Z.Yu Li, H.Jiang & H.Xu) Mich. Möller & A.Weber, *P. minutimaculata* (D. Fang & W. T. Wang) Yin Z.Wang, *P. obtusidentata* (W.T. Wang) Mich. Möller & A.Weber and *P. tribracteata* (W.T. Wang) Mich. Möller & A.Weber (e.g. Wang et al. 1998; Wei et al. 2010; Pan et al. 2013; Shui et al. 2017; Wen et al. 2019). Within the above complex, the new species is different from all the others in its white throat outside the corolla, except for *P. lungzhouensis*. Furthermore, the new species is different from *P. lungzhouensis* in its bracts 1.6–2.5 × 1.3–1.5 cm, corolla 3–3.5 cm long, corolla tube slightly curved near the base, inflated on the adaxial surface near the corolla lobes and corolla abaxial lip lobes rounded. *P. lungzhouensis*, however, has bracts 2.5–3.8 × 2.2–2.8 cm, corolla 4.5–5 cm long, corolla tube not curved, not inflated on the adaxial surface near the corolla lobes and corolla abaxial lip lobes triangular. In a word, the new species is unique in its narrow and curved corolla tube amongst the above complex with prominent bracts (Table 2).

**Table 2.** Morphological comparison of *Primulina xuansonensis* W.H. Chen & Y. M. Shui and *P. lungzhouensis* (W.T. Wang) Mich.Möller & A.Weber.

**Characters**	***Primulina xuansonensis***	***P. lungzhouensis***
Bracts	1.6–2.5 × 1.3–1.5 cm	2.5–3.8 × 2.2–2.8 cm
Corolla	3–3.5 cm long, ca. 0.8 cm wide at the throat	4.5–5 cm long, ca. 1.4 cm wide at the throat
Corolla tube	Slightly curved near the base, inflated on the adaxial surface near the corolla lobes	Not curved, not inflated on the adaxial surface near the corolla lobes
Abaxial lip lobes of corolla	Rounded	Triangular

## Supplementary Material

XML Treatment for Paraboea sinensis var. glabrissima

XML Treatment for Primulina xuansonensis
